# Characterization of unique pattern of immune cell profile in patients with nasopharyngeal carcinoma through flow cytometry and machine learning

**DOI:** 10.1111/jcmm.18404

**Published:** 2024-06-18

**Authors:** Li‐Jen Liao, Chien‐Chen Tsai, Po‐Yu Li, Cheng‐Yun Lee, Shian‐Ren Lin, Wan‐Yu Lai, I‐Yu Chen, Chiung‐Fang Chang, Jan‐Mou Lee, Yen‐Ling Chiu

**Affiliations:** ^1^ Department of Otolaryngology Far Eastern Memorial Hospital New Taipei City Taiwan; ^2^ Department of Anatomical Pathology Far Eastern Memorial Hospital New Taipei City Taiwan; ^3^ FullHope Biomedical Co., Ltd New Taipei City Taiwan; ^4^ Division of Nephrology, Department of Medicine Far Eastern Memorial Hospital New Taipei City Taiwan; ^5^ Department of Medical Research Far Eastern Memorial Hospital New Taipei City Taiwan; ^6^ Graduate Institute of Medicine and Graduate Program in Biomedical Informatics Yuan Ze University Taoyuan Taiwan; ^7^ Graduate Institute of Clinical Medicine National Taiwan University College of Medicine Taipei Taiwan

**Keywords:** flow cytometry, immune cell profile, machine learning, nasopharyngeal carcinoma (NPC)

## Abstract

In patients with nasopharyngeal carcinoma (NPC), the alteration of immune responses in peripheral blood remains unclear. In this study, we established an immune cell profile for patients with NPC and used flow cytometry and machine learning (ML) to identify the characteristics of this profile. After isolation of circulating leukocytes, the proportions of 104 immune cell subsets were compared between NPC group and the healthy control group (HC). Data obtained from the immune cell profile were subjected to ML training to differentiate between the immune cell profiles of the NPC and HC groups. We observed that subjects in the NPC group presented higher proportions of T cells, memory B cells, short‐lived plasma cells, IgG‐positive B cells, regulatory T cells, MHC II^+^ T cells, CTLA4^+^ T cells and PD‐1^+^ T cells than subjects in the HC group, indicating weaker and compromised cellular and humoral immune responses. ML revealed that monocytes, PD‐1^+^ CD4 T cells, memory B cells, CTLA4^+^ CD4 T_reg_ cells and PD‐1^+^ CD8 T cells were strongly contributed to the difference in immune cell profiles between the NPC and HC groups. This alteration can be fundamental in developing novel immunotherapies for NPC.

## 1 INTRODUCTION

In 2020, nasopharyngeal carcinoma (NPC) was the 24th most common and lethal cancer worldwide, accounting for 133,000 newly diagnosed cases (0.7% of all new cancer cases) and 80,000 cancer‐related deaths (0.8% of all cancer‐related deaths).^1^ Because NPC is associated with various subjective symptoms, approximately 75.4% of newly diagnosed cases are discovered at an advanced stage (stage III or IV), and 15% to 30% of patients with advanced NPC develop recurrent or metastatic (R/M) disease after their initial curative treatment.^2^ Although over 80% of patients with R/M NPC benefit from conventional treatment options, such as intensity‐modulated radiotherapy combined with chemotherapy, 10% of these patients develop radioresistant diseases.^3^, ^4^, ^5^, ^6^ Therefore, immune checkpoint inhibitor (ICI) immunotherapy has emerged as a viable treatment option for these patients. In ICI immunotherapy, the clinical responses of patients with NPC are assessed using factors such as tumour mutation burden or the intratumoral expression of programmed cell death ligand‐1 (PD‐L1).^7^, ^8^ Nevertheless, the overall response rates of R/M NPC to ICI immunotherapy fall between 18% and 26%, indicating that utilizing the intratumoral expression of PD‐L1 or tumour mutation burden in assessing the clinical response of R/M NPC to ICI immunotherapy is insufficient.^9^ Therefore, further tool is required to precisely estimate the clinical response of R/M NPC to ICI immunotherapy.

Alterations in immune cell profile often occur during the progression of various cancer types, thereby potentially guiding physicians in the precise evaluation of clinical responses to ICI immunotherapy in patients with cancer.^10^, ^11^ In patients with NPC, the immune cell profile in peripheral blood remains unclear. Therefore, in this study, we comprehensively evaluated immune cell profile alterations in peripheral blood in patients with NPC. We isolated leukocytes from both patients with NPC and healthy controls (HCs) and identified 104 circulating immune cell subsets from the given leukocytes. These subsets were subjected to pairwise comparisons for the NPC and HC groups. To further clarify the aforementioned alterations, we used machine learning (ML) to identify the immune cell subsets that considerably influenced these alterations in immune cell profiles.

## 2 METHODS

### 2.1 Study design and patient enrolment

In this trial, our primary objective was to elucidate the alterations in the immune cell profiles of patients with NPC. To this end, we isolated leukocytes from patients with NPC and age‐matched HCs and compared the proportions of immune cell subsets in leukocytes belong to the two populations. Both patients with NPC and HCs participated in this trial through investigator invitations and open recruitment at Far Eastern Memorial Hospital (New Taipei, Taiwan) from September 2020 to April 2023. Patients with NPC meeting the following criteria were included: receiving a recent diagnosis of NPC in accordance with practice guidelines; having no central nervous system metastasis; not having received any NPC‐targeted therapy (either conventional or investigational) within 1 month before enrolment; and testing negative for human immunodeficiency virus and *Treponema pallidum*. Patients with NPC who had any severe and extensive cardiac, pulmonary, hepatic or nephrological disorders were excluded. All HCs met the same inclusion criteria as those of the NPC group except for the criterion stipulating an NPC diagnosis. After each patient signed an informed consent form, 20 mL of peripheral blood was collected for analysis.

This study was conducted in accordance with the Declaration of Helsinki. The study protocol was approved by the Institutional Review Board of Far Eastern Memorial Hospital (approval no. 108170‐E on 4 February 2020).

### 2.2 Reagents, antibodies and software

Tables S1 and S2 list the reagents and antibodies used in this study. Once received, all reagents and antibodies were aliquoted and stored in accordance with the manufacturers' instructions until use. ML training was conducted using the *scikit‐learn* Python module, which is available on GitHub.

### 2.3 Isolation of peripheral blood mononuclear cells and leukocytes

The blood samples were divided into two groups. The first group underwent density centrifugation to obtain peripheral blood mononuclear cells (PBMCs) for lymphocyte, monocyte and lineage cell identification,^12^ and the second group underwent isotonic erythrocyte lysis to obtain leukocytes for granulocyte identification. Subsequently, the leukocytes and PBMCs were suspended in a staining buffer (0.5% bovine serum albumin and 0.02% sodium azide in phosphate‐buffered saline) and subjected to immunostaining.

### 2.4 Immunostaining and flow cytometry

Details regarding the immunostaining protocol were as shown before.^12^ After staining, we analysed cell fluorescence by using a Navios flow cytometer (Beckman Coulter, Brea, CA, USA) coupled with Kaluza Analysis Software version 1.3 (Beckman Coulter) for data acquisition.

### 2.5 Data acquisition, pairwise comparison and ML

Immune cell subsets were identified using Kaluza Analysis Software following the definitions outlined in Table S3 through the filtration pedigrees illustrated in Figures S1–S5. The number of immune cell subsets was presented as the ratio between each immune cell subset and its corresponding parent cell subset.

The immune cell profiles of the NPC and HC groups were compared in a pairwise manner and an ML model. In this pairwise comparison, the number of immune cell subsets belonging to the NPC and HC groups was directly compared.

**FIGURE 1:**
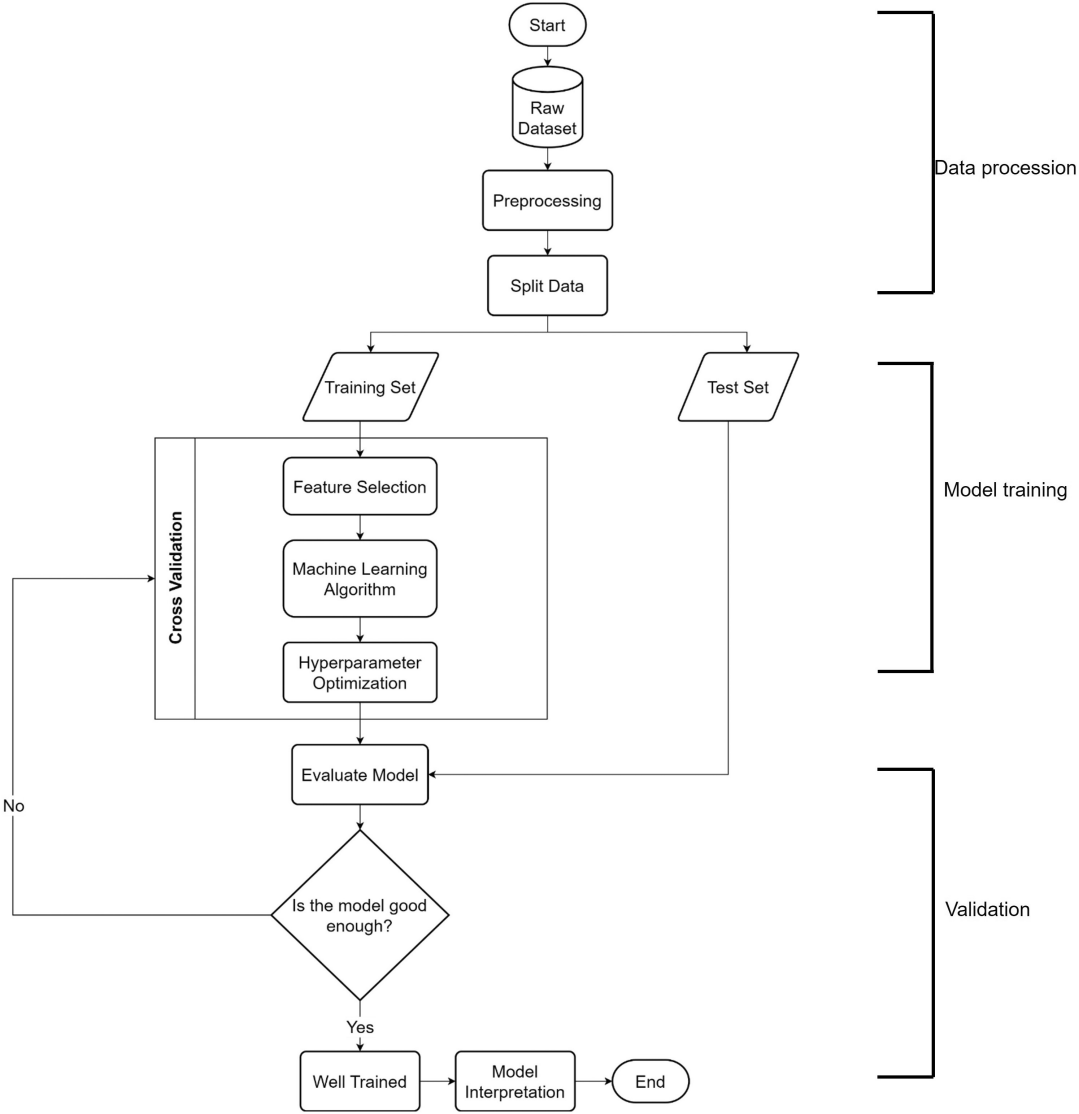
Schematic illustration of machine learning (ML) model training. The detailed process of machine learning was available in Materials and Methods. Briefly, correlation among the immune cell subsets was calculated, and those complementary immune cell subset pairs with high correlation were trimmed. Then, we randomly assigned data from 7 nasopharyngeal carcinoma (NPC) patients and 10 healthy controls (HCs) to the tested data set and kept away from machine learning until the validation step. The remaining immune cell subsets were proceeded to feature selection and machine learning with cross‐validation. After model training, the predictive capabilities of the trained model were determined by receiver operating characteristic curve (ROC) analysis, and the contribution of the immune cell subsets in discriminating data of NPC patients and HCs was interpreted by the Shapley Additive exPlanations (SHAP) explanation method.

As shown in Figure 1, our ML analysis involved the three steps of data processing, model training and model validation. During data processing, complementary immune cell subset pairs (hereinafter referred to as features) with a high correlation (Pearson's coefficient of correlation ≥0.8) were trimmed. Subsequently, data obtained from 7 patients with NPC and 10 HCs were used for model validation, and the remaining data were used for feature selection with the Boruta algorithm following ML training with random forest classification. During model training, repeated stratified *k*‐fold cross‐validation and Optuna hyperparameter search were used for cross‐validation and hyperparameter search, respectively. After model training, the predictive capabilities of the trained model were evaluated using both a confusion matrix and the area under the receiver operating characteristic (ROC) curve. Finally, the SHapley Additive exPlanations (SHAP) explanation method was used to analyse the contribution of each feature to NPC and HC discrimination.

### 2.6 Statistical analysis

The results of pairwise comparisons are presented as means ± standard deviations on plots created by GraphPad Prism version 9 (GraphPad Software, La Jolla, CA, USA). The statistical significance of each pairwise comparison was calculated using the Mann–Whitney *U* test. Features with significant differences were plotted on bar charts with *, **, *** and **** indicating *p* values smaller than 0.05, 0.01, 0.001 and 0.0001, respectively.

## 3 RESULTS

### 3.1 Patient demographics

Table 1 presents the demographic characteristics of the study patients. A total of 24 patients with NPC and 33 HCs, with a median age of 48.5 (range: 37–79) and 44 (range: 25–74) years, respectively, participated in the trial. The majority of patients in the NPC group were at stage II (10 patients), followed by stage III (5 patients) and stage IV (4 patients). A histological examination revealed that 20 out of the 24 patients with NPC had a nonkeratinized and undifferentiated subtype of NPC (World Health Organization/International Agency for Research on Cancer classification type 3). Notably, 15 out of the 24 patients with NPC were seropositive for Epstein–Barr virus (EBV) viral capsid antigen immunoglobulin A (two with a grey‐zone value), whereas only two patients were found to carry the EBV, indicating the past remission of EBV infection in patients with NPC.^13^

**TABLE 1:** Patient demographics.

Characteristics	NPC	HC	*p*‐value
	*N* = 24	*N* = 33	
Female, *N* (%)	11 (45.8)	17 (48.6)	
Median age, year (range)	48.5 (37–79)	44 (25–74)	0.5707
Stage, *N* (%)			
I	4 (16.7)		
II	10 (41.7)		
III	5 (20.8)		
IV	5 (20.8)		
WHO/IARC classification, *N* (%)			
II	3 (12.5)		
III	20 (83.3)		
Unknown	1 (4.2)		
EBV VCA IgA, *N* (%)			
Positive	13 (54.2)		
Negative	9 (37.5)		
Grey‐zone	2 (8.3)		

Abbreviations: HC, healthy control; NPC, nasopharyngeal carcinoma.

### 3.2 Establishment of an immune cell profile

As shown in Table S3, the immune cell profile established in this study comprised 104 features encompassing various aspects of the immune system, including innate immunity (granulocytes, natural killer [NK] cells, NKT cells, dendritic cells and monocytes), cellular immunity (T‐cell lineage), humoral immunity (B‐cell lineage) and immune tolerance (PD‐L1^+^ cells, PD1^+^PD‐L1^+^ cells, regulatory cells and PD‐1^+^ regulatory cells). From this comprehensive set of features, we identified 23 features whose quantities exhibited significant differences between the NPC and HC groups. In the following sections, we describe our findings.

### 3.3 Patients with NPC exhibit dysregulated immunity

**FIGURE 2:**
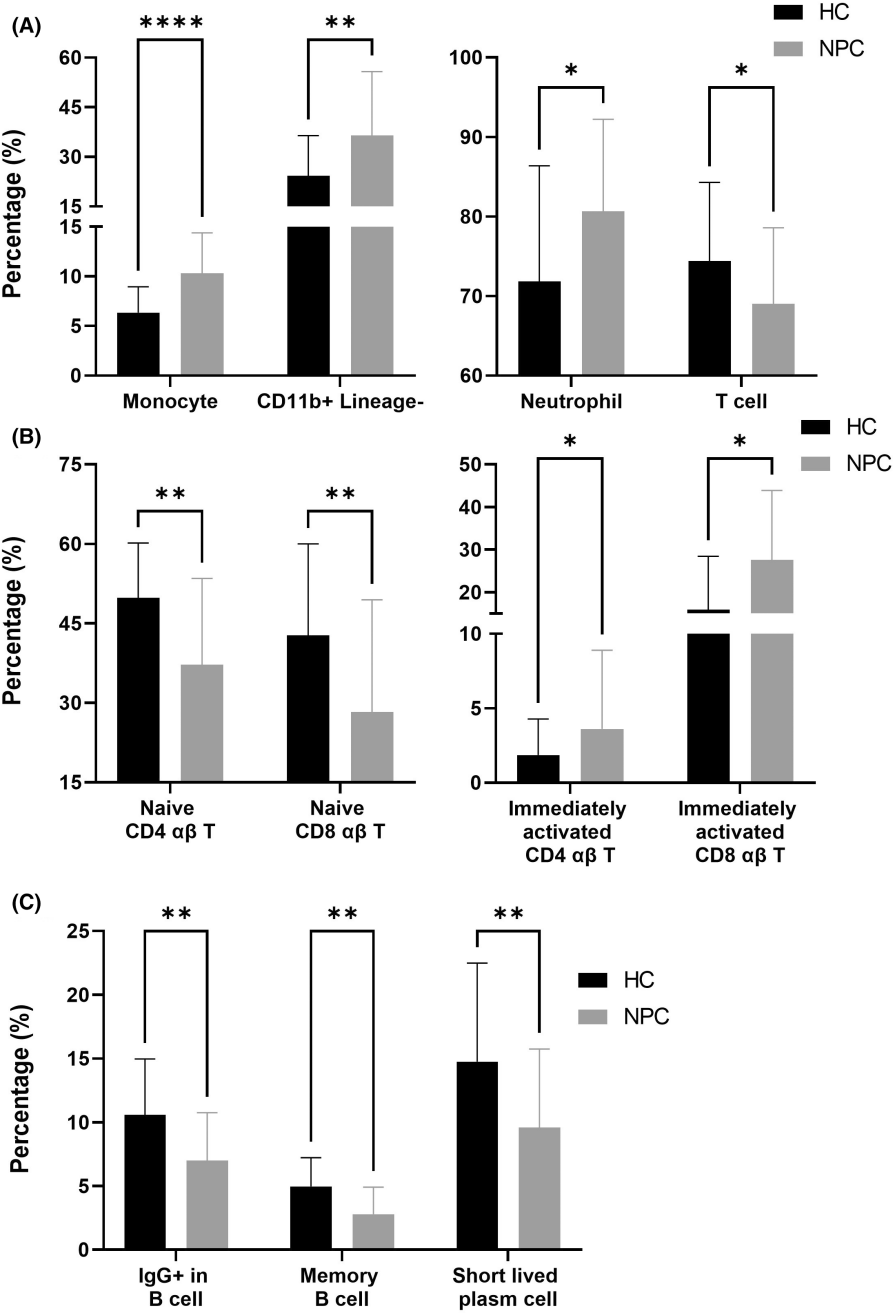
NPC patients exhibited dysregulation in effective immunity. The process of immunoprofiling analysis was available in Materials and Methods. The proportion of immune cell subsets between NPC patients and HCs was compared pairwisely, and those immune cell subsets belonging to (A) lineage cells and granulocytes, (B) T‐cell lineage and (C) B‐cell lineage with significantly proportional differences between NPC patients and HCs were plotted. Data were shown in mean ± standard deviation with *, ** and **** labelling while *p*‐value (computed by Mann–Whitney *U* test) of the comparison was lower than 0.05, 0.01 and 0.0001, respectively.

Granulocytes, monocytes, dendritic cells, NK cells and NKT cells are effector cells involved in innate immunity.^14^ In terms of innate immunity, we identified a significantly larger number of monocytes and CD11b^+^Lineage^−^ cells in the NPC group than in the HC group (*p* < 0.0001 [monocytes] and 0.0096 [CD11b^+^Lineage^−^ cells], Figure 2A), which was attributable to the significantly larger number of neutrophils observed in the NPC group (*p* = 0.0338, Figure 2A).

In terms of cellular immunity, we identified a significantly smaller number of total T cells in the NPC group than in the HC group (*p* = 0.0338, Figure 2A), which was attributable to the significantly smaller number of naïve CD4 and CD8 αβ T cells observed in the NPC group (*p* = 0.0048 [naïve CD4 αβ T cell] and 0.0048 [naïve CD8 αβ T], Figure 2B). Compared with the number of naïve T cells, the numbers of effector memory and central memory T cells were higher in the NPC group (data not shown). In addition, the number of immediately activated αβ T cells (both CD4 and CD8 αβ T cells) was significantly higher in the NPC group than in the HC group (*p* = 0.0142 [immediately activated CD4 T] and 0.0142 [immediately activated CD8 T], Figure 2B). Compared with effector T cells, immediately activated T cells are more active in interferon‐γ secretion,^15^ which may drive the activation of cellular immunity.^16^ These results indicated that the cellular immunity of the NPC group was activated, but the number of effector cells was potentially insufficient.

In terms of humoral immunity, we identified a significantly smaller number of IgG^+^ B cells in the NPC group compared with the HC group (*p* = 0.0048, Figure 2C), which was attributable to the significantly small number of memory B cells observed in the NPC group (*p* = 0.0011, Figure 2C). We also identified a significantly smaller number of short‐lived plasma cells in the NPC group compared with the HC group (*p* = 0.0048, Figure 2C). Memory B cells and short‐lived plasma cells play a key role in humoral immunity.^17^ In patients with NPC, a reduction in the numbers of these cells indicates compromised humoral immunity.

### 3.4 Patients with NPC exhibit upregulated immune tolerance

**FIGURE 3:**
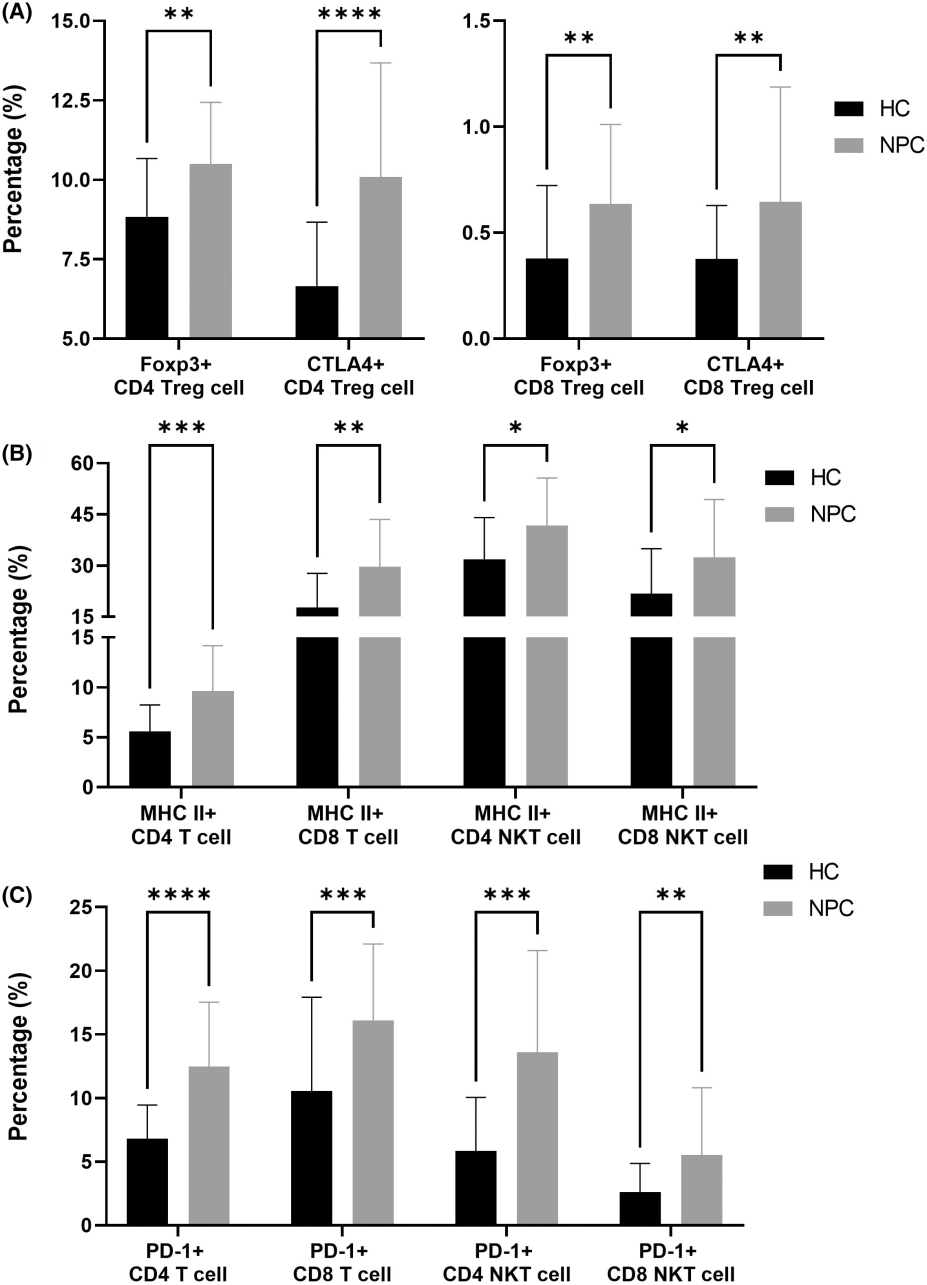
NPC patients displayed upregulated immune tolerance. The process of immunoprofiling analysis was available in Materials and Methods. The proportion of immune cell subsets between NPC patients and HCs was compared pairwisely, and those immune cell subsets belonging to (A) regulatory cells, (B) MHC II‐positive cells and (C) PD‐1‐positive cells with significantly proportional differences between NPC patients and HCs were plotted. Data were shown in mean ± standard deviation with *, **, *** and **** labelling while *p*‐value (computed by Mann–Whitney *U* test) of the comparison was lower than 0.05, 0.01, 0.001 and 0.0001, respectively.

In terms of regulatory cells, we identified a significantly larger number of FoxP3^+^ T_reg_ (both CD4 and CD8) and CTLA4^+^ T_reg_ (both CD4 and CD8) cells in the NPC group than in the HC group (*p* = 0.0021 [Foxp3^+^ T_reg_ CD4 T], 0.0022 [Foxp3^+^ T_reg_ CD8 T], <0.0001 [CTLA4^+^ T_reg_ CD4 T] and 0.0062 [CTLA4^+^ T_reg_ CD8 T], Figure 3A). FoxP3^+^ T_reg_ cells postpone the activation of T cells by increasing the circulating levels of IL‐10,^18^ and CTLA4^+^ T_reg_ cells raise the threshold of T‐cell receptor and maintain the activation of T cells.^19^ In patients with NPC, increases in the numbers of FoxP3^+^ or CTLA4^+^ T_reg_ cells indicate the postponement of T‐cell activation.

Compared with the HC group, the NPC group had a significantly larger number of MHC II^+^ T cells (both CD4 and CD8) and MHC II^+^ NKT cells (both CD4 and CD8; *p* = 0.0005 [MHC II^+^ CD4 T], 0.0019 [MHC II^+^ CD8 T], 0.0214 [MHC II^+^ CD4 NKT] and 0.0214 [MHC II^+^ CD8 NKT], Figure 2B). MHC II‐expressed CD8 T cells are frequently co‐expressed with PD‐1.^20^ In addition, MHC II^+^ CD4 T cells play a key role in persistent infections with human immunodeficiency virus,^21^ indicating that MHC II‐expressed T cells potentially engage in immune evasion.

Compared with the HC group, the NPC group had a significantly larger number of PD‐1^+^ T cells (both CD4 and CD8) and PD‐1^+^ NKT cells (both CD4 and CD8; *p* ≤ 0.0001 [PD‐1^+^ CD4 T], 0.0010 [PD‐1^+^ CD8 T], 0.0002 [PD‐1^+^ CD4 NKT] and 0.0060 [PD‐1^+^ CD8 NKT], Figure 3C). The interaction between PD‐1 and PD‐L1 triggers T‐cell exhaustion.^22^ In patients with NPC, an increase in the number of PD‐1^+^ T cells indicates T‐cell exhaustion.

### 3.5 Monocytes are the hallmark of immune cell profile alterations in patients with NPC

To determine the effect of the proportions of different features on immunity, we used ML to elucidate the contribution of these features to the distinction between the NPC and HC groups. Before we input the data for ML, we trimmed complementary immune cell‐subset pairs with a high correlation (|*r*| ≥ 0.8) and subjected the remaining data (78 features in total) to ML training. Subsequently, we randomly assigned the data collected from 7 patients with NPC and 10 HCs to a validation data set, which was used to verify the predictive performance of the ML model.

**FIGURE 4:**
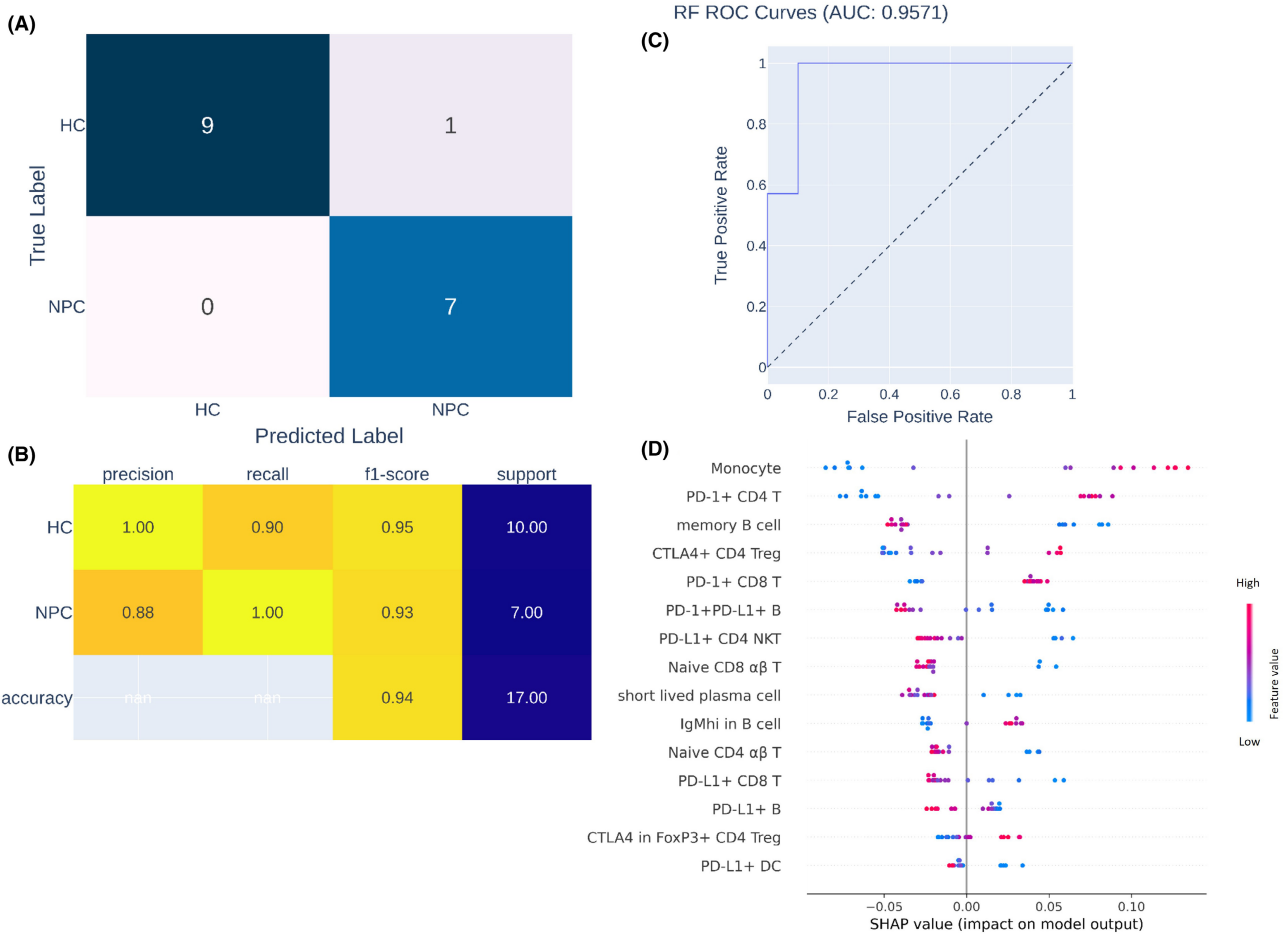
Trained ML model can well distinguish data of NPC patients from HCs. The process of ML training was illustrated in Figure 1. After model training, the predictive performance of ML model was tested by the test data set and shown in (A) confusion matrix, (B) classification report and (C) ROC analysis. (D) To evaluate the individual contribution of each selected immune cell subsets in discriminating data from NPC patients and HCs, SHAP explanation method was performed and represented in beeswarm plot.

To identify the hallmark of the immune cell profiles of patients with NPC, we trained an ML algorithm to differentiate between the immune cell profiles of patients with NPC and HCs (Figure 1), and we also examined the contribution of each feature to such differentiation. During ML training, 15 highly effective differentiating features were identified. After model training, untrained data obtained from 7 patients with NPC and 10 HCs were loaded to the trained ML model to evaluate its predictive capability and area under the curve (AOC). As shown in Figure 4A,B, the data obtained from seven patients with NPC and nine HCs were correctly identified by the trained ML model, with a precision, recall, and F1‐score of 1.00, 0.90 and 0.95, respectively. Therefore, given the results of our ROC analysis (AOC = 0.9571, Figure 4C), our trained ML model can precisely differentiate between the data of patients with NPC and HCs. According to the SHAP explanation results, the five most highly ranked features for NPC and HC differentiation were monocytes, PD‐1^+^ CD4 T cells, memory B cells, CTLA4^+^ CD4 T_reg_ cells and PD‐1^+^ CD8 T cells, the levels of which significantly differed between the NPC and HC groups. In summary, according to the results of our ML model, the immune cell profiles of patients with NPC are characterized by differences in monocytes, PD‐1^+^ CD4 T cells, memory B cells, CTLA4^+^ CD4 T_reg_ cells and PD‐1^+^ CD8 T cells relative to those of non‐NPC patients.

## 4 DISCUSSION

In this study, we extensively investigated the differences in the immune cell profiles of patients with NPC and HCs through pairwise comparisons of the proportions of certain features. Our results indicated that the NPC group had a small number of naïve T cells and B‐cell lineages, indicating the activation of their cellular immunity and reduced humoral immunity. In addition, we observed increased immune tolerance in the NPC group. Using an ML model, we discovered that monocytes, PD‐1^+^ CD4 T cells, memory B cells, CTLA4^+^ CD4 T_reg_ cells and PD‐1^+^ CD8 T cells contributed to alterations in immune cell profiles.

Adjustment of the profile of circulating immune cells is well‐documented in various types of cancer.^23^, ^24^ For example, in non‐small‐cell lung cancer, immune cell profile alterations are believed to be a promising biomarker for predicting clinical responses to ICI immunotherapy.^25^ Although previous studies have well‐documented the features of immune cell profile in the tumour microenvironment of NPC,^26^, ^27^, ^28^ characterization of circulating immune cell profiling remains unclear. In this study, we examined the requirements of circulating immune cell profiling in patients with NPC and provided comprehensive treatment‐related information.

In this study, we observed a significant reduction in the number of total T cells in the NPC group (Figure 2A). However, we observed an increase in the number of immune cell subsets associated with the activation of cellular immunity (Figure 2B). These results indicated that patients with NPC had active but insufficient cellular immunity. Generally, the expression of PD‐L1 in tumours positively correlates with the number of PD‐1^+^ T cells in peripheral blood.^29^ Therefore, patients with NPC who exhibit high levels of PD‐L1 can be treated with PD‐1 or PD‐L1 ICIs. Nevertheless, according to the NCI‐9742 and POLARIS‐02 clinical trials, less than half of patients with PD‐L1^+^‐expressing NPC respond to anti‐PD‐1 ICIs.^30^, ^31^ Given the insufficiency of T cells in the current study, patients with PD‐L1‐expressing NPC who do not respond to anti‐PD‐1 ICIs may experience T‐cell insufficiency. To address this insufficiency, the adoptive transfer of tumour‐specific cytotoxic T lymphocytes (CTLs) may aid in the control of tumours in patients with NPC. In EBV‐endemic areas, over 90% of patients with NPC present with EBV infection,^32^ indicating the potential benefit of utilizing EBV‐specific CTLs (EBV‐CTLs) for NPC control. In a clinical trial, Straathof et al.^33^ used autologous EBV‐specific cytotoxic T cells to treat relapsed patients with advanced EBV‐associated NPC. They reported that four out of six patients experienced controlled disease, with an objective response rate of 50%. In another study, Comoli et al.^34^ used EBV‐CTLs to treat patients with stage IV EBV‐associated NPC. They reported that one out of five patients with NPC experienced partial remission, and two out of five patients with NPC remained stable. These results underscore the potential of EBV‐CTL adoptive transfer in treating patients with EBV‐associated NPC.

In addition to T‐cell sufficiency, we observed elevated immune tolerance in the NPC group, which was associated with an increase in the number of PD‐1^+^ T cells, CTLA4^+^ T cells, MHC II^+^ T cells and T_reg_ cells in the NPC group (Figure 3), thus potentially diminishing the clinical benefits of tumour‐specific cytotoxic T cells.^35^ In a clinical trial, Huang et al.^36^ transferred autologous EBV‐CTLs to 28 patients with R/M NPC and evaluated their clinical response. They reported that only one patient benefited from this treatment. In another clinical trial, Louis et al.^37^ transferred autologous EBV‐CTLs to 25 patients with locoregional refractory NPC and evaluated their clinical response. They reported that 17 patients benefited from this treatment. These findings indicate that utilizing EBV‐CTL monotherapy for the treatment of NPC may be insufficient to limit the progression of NPC. According to our results, a combination therapy of anti‐PD‐1 or anti‐CTLA4 ICIs and EBV‐CTLs may yield favourable clinical outcomes for patients with NPC. Smith et al.^38^ reported a case in which a sequential treatment with EBV‐CTLs and nivolumab was used, with complete remission after treatment. This case report supports our hypothesis regarding the clinical benefits of combining EBV‐CTLs and ICIs for patients with NPC.

In this study, we discovered that the number of monocytes was significantly higher in the NPC group than in the HC group (Figure 2A). In NPC, tumour cells secrete a granulocyte macrophage colony‐stimulating factor to recruit the circulating monocytes accumulating in the tumour site and cause them to differentiate into tumour‐associated macrophages, thereby promoting NPC metastasis by inducing the epithelial–mesenchymal transition of NPC through upregulated proinflammatory cytokines.^39^, ^40^, ^41^ We also observed that the number of circulating monocytes was higher in the NPC group than in the other groups. According to Kiss et al.,^42^ the number of circulating monocytes increases as a result of either increased monocyte mobilization from the bone marrow or elevated monopoiesis. Jin et al.^43^ reported a significant increase in the level of serum C‐C motif chemokine 2 (CCL2, also known as monocyte chemoattractant protein 1) in patients with NPC. They indicated that this increase in the level of serum CCL2 positively correlated with the progression of NPC, indicating that NPC tumours secrete CCL2 to recruit monocytes from the bone marrow.

## 5 CONCLUSION

In this study, we established an immune cell profile for patients with NPC from peripheral blood and examined the characteristics of this profile by comparing the immune cell profiles of patients with NPC and HCs. Overall, our immune cell profile can serve as a valuable reference for future studies evaluating pivotal immunotherapies for NPC. In the future, we intend to further enhance our immune cell profile and identify other predictive biomarkers of ICI immunotherapy for patients with NPC.

## AUTHOR CONTRIBUTIONS

**Li‐Jen Liao:** Investigation (lead); writing – original draft (equal). **Chien‐Chen Tsai:** Investigation (equal); writing – original draft (equal). **Po‐Yu Li:** Investigation (equal). **Cheng‐Yun Lee:** Formal analysis (equal). **Shian‐Ren Lin:** Writing – original draft (equal). **Wan‐Yu Lai:** Investigation (equal). **I‐Yu Chen:** Investigation (equal). **Chiung‐Fang Chang:** Writing – original draft (equal); writing – review and editing (equal). **Jan‐Mou Lee:** Conceptualization (equal); funding acquisition (equal); project administration (equal); writing – review and editing (equal). **Yen‐Ling Chiu:** Conceptualization (equal); project administration (equal); writing – review and editing (equal).

## FUNDING INFORMATION

This work was supported by Far Eastern Memorial Hospital (grant number: FEMH‐2020‐C‐009 and FEMH‐2021‐C‐009) to Dr. Yen‐Ling Chiu. This equipment, reagent and cost associated with immune phenotyping and machine learning were fully sponsored by FullHope Biomedical Co., Ltd.

## CONFLICT OF INTEREST STATEMENT

The authors claim no conflict of interest in this study.

## PATIENT CONSENT STATEMENT

Informed consent was obtained from all individual participants included in the study.

## Supporting information


**Data S1.** Supporting Information.

## Data Availability

The datasets generated during and/or analysed during the current study are available from the corresponding author on reasonable request.
