# Supplementary material for: Characterization of unique pattern of immune cell profile in patients with nasopharyngeal carcinoma through flow cytometry and machine learning
Source: J Cell Mol Med. 2024 Jun 18;28(12):e18404. doi: 10.1111/jcmm.18404 (PMC11184936; doi:10.1111/jcmm.18404)
Supplement: Supplementary file 1 — Data S1. Supporting Information. [file JCMM-28-e18404-s001.docx]

**Supplementary Table 1. Reagents utilized in this study**

| **Reagent** | **Manufacture** |
| --- | --- |
| RBC lysis buffer | Biolegend |
| Bovine serum albumin | Merck |
| Ficoll-Paque^TM^ medium (Density 1.077 g/mL) | Thermo-Fisher |
| Foxp3/Transcription Factor Staining Buffer Set | Thermo-Fisher |
| Sodium azide | Merck |

**Supplementary Table 2. Antibodies applied in this study**

| **Targets** | **Clone** | **Fluorophore** | **Manufacture** |
| --- | --- | --- | --- |
| CCR7 | G043H7 | PE/Cy7 | BioLegend |
| CD3 | UCHT1 | KO | Beckman-Coulter |
| CD4 | SFCI12T4D11 | PE/Cy7 | Beckman-Coulter |
| CD8 | B9.11 | KO | Beckman-Coulter |
| CD10 | HI10a | PE/Cy7 | BioLegend |
| CD11b | Bear1 | PE/Cy7 | Beckman-Coulter |
| CD11c | 3.9 | APC | BioLegend |
| CD14 | RMO52 | APC/AF750 | Beckman-Coulter |
| CD16 | 3G8 | KO | Beckman-Coulter |
| CD19 | J3-119 | APC/AF750 | Beckman-Coulter |
| CD21 | Bu32 | APC | BioLegend |
| CD23 | EBVCS-5 | PE | BioLegend |
| CD25 | BC96 | PE | BioLegend |
| CD27 | M-T271 | PB | BioLegend |
| CD28 | CD28.2 | PE/Cy7 | BioLegend |
| CD38 | HB-7 | PerCP/Cy5.5 | BioLegend |
| CD45 | J33 | ECD | Beckman-Coulter |
| CD45RO | UCHL1 | APC | BioLegend |
| CD56 | N901 | APC/AF700 | Beckman-Coulter |
| CD64 | 10.1 | AF700 | BioLegend |
| CD66b | G10F5 | PB | BioLegend |
| CD69 | FN50 | PB | BioLegend |
| CD123 | 6H6 | PE | BioLegend |
| CD127 | A019D5 | BV421 | BioLegend |
| CD193 | 5E8 | FITC | BioLegend |
| FoxP3 | 29E.2A3 | APC | Thermo-Fisher |
| LAG-3 | 11C3C65 | PerCP/Cy5.5 | BioLegend |
| MHC II (HLA-DR) | Immu-357 | PB | Beckman-Coulter |
| PD-1 | IP26 | PerCP/Cy5.5 | BioLegend |
| PD-L1 | B1 | PE | BioLegend |
| TCRγ/δ | 236A/E7 | APC | BioLegend |
| TCRα/β | L3D10 | FITC | BioLegend |
| TIM-3 | F38-2E2 | PB | BioLegend |
| ***Isotype control*** | | | |
| IgG | G18-145 | BV510 | BD Biosciences |
| IgM | MHM-88 | AF700 | BioLegend |

**Abbreviation:** AF700, Alexa fluor^®^ 700; APC, allophycocyanin; BV421, brilliant violet 421; Cy5.5, cyanine 5.5; ECD, electron coupled dye; FITC, fluorescein isothiocyanate; KO, krome orange; PB, pacific blue; PE, phycoerythrin; PerCP, peridinin-chlorophyll-protein.

**Supplementary Table 3. Definition of immune cell subsets with markers**

| **Cell name** | **Markers** |
| --- | --- |
| ***Granulocytes*** |  |
| CD11b^+^ Lineage^-^ cell | CD3^-^CD14^-^CD19^-^CD56^-^CD11b^+^ |
| Neutrophil | CD3^-^CD14^-^CD19^-^CD56^-^CD11b^+^CD16^+^CD64^-^CD66b^+^CD123^-^CD193^-^ |
| Eosinophil | CD3^-^CD14^-^CD19^-^CD56^-^CD11b^+^CD16^-^CD66b^+^CD123^-^CD193^+^ |
| Basophil | CD3^-^CD14^-^CD19^-^CD56^-^CD11b^+^CD16^-^CD66b^-^CD123^+^CD193^+^ |
| ***NK cell*** |  |
| NK | CD3^-^CD14^-^CD19^-^CD56^+^ |
| CD8 NK | CD3^-^CD14^-^CD19^-^CD56^+^CD4^-^CD8^+^ |
| DN NK | CD3^-^CD14^-^CD19^-^CD56^+^CD4^-^CD8^-^ |
| ***NKT cell*** |  |
| NKT | CD3^+^CD14^-^CD19^-^CD56^+^ |
| CD4 NKT | CD3^+^CD14^-^CD19^-^CD56^+^CD4^+^ |
| CD8 NKT | CD3^+^CD14^-^CD19^-^CD56^+^CD4^-^ |
| ***Dendritic cell*** |  |
| DC | CD3^-^CD14^-^CD19^-^CD56^-^CD11c^+^ |
| MHC II^+^ DC | CD3^-^CD14^-^CD19^-^CD56^-^CD11c^+^MHC II^+^ |
| ***B cell*** |  |
| B cell | CD14^-^CD19^+^ |
| IgG^+^ in B cell | CD19^+^CD127^-^IgG^+^IgM^-^ |
| Long lived plasma cell | CD19^+^CD127^-^IgG^+^IgM^-^CD10^+^CD21^+^ |
| Germinal center B cell | CD19^+^CD127^-^IgG^+^IgM^-^CD10^+^CD21^-^ |
| Memory B cell | CD19^+^CD127^-^IgG^+^IgM^-^CD10^-^CD21^+^CD23^-^CD38^+^ |
| IgM^dim^ in B cell | CD19^+^CD127^-^IgG^-^IgM^dim^ |
| Follicular B cell | CD19^+^CD127^-^IgG^-^IgM^dim^CD21^+^CD23^+^CD38^+^CD10^-^ |
| Short lived plasma cell | CD19^+^CD127^-^IgG^-^IgM^dim^CD21^+^CD23^-^CD38^+^CD10^-^ |
| IgM^hi^ in B cell | CD19^+^CD127^-^IgG^-^IgM^hi^ |
| Marginal Zone B cell | CD19^+^CD127^-^IgG^-^IgM^hi^CD21^+^CD10^-^CD38^+^CD23^-^ |
| Transitional B cell | CD19^+^CD127^-^IgG^-^IgM^hi^CD21^-^CD10^+^CD38^+^CD23^-^ |
| MHC II^+^ B cell | CD14^-^CD19^+^MHC II^+^ |
| ***Monocyte*** |  |
| Monocyte | CD14^+^CD19^-^ |
| MHC II^+^ monocyte | CD14^+^CD19^-^MHC II^+^ |
| ***T lymphocyte*** |  |
| T cell | CD3^+^CD14^-^CD19^-^CD56^-^ |
| CD4 αβ T | CD3^+^CD14^-^CD19^-^CD56^-^TCR_αβ_^+^TCR_γδ_^-^CD4^+^CD8^-^ |
| Terminal effector  CD4 αβ T | CD3^+^CD14^-^CD19^-^CD56^-^TCR_αβ_^+^TCR_γδ_^-^CD4^+^CD8^-^CD25^-^CD69^+^ |
| Immediately activated  CD4 αβ T | CD3^+^CD14^-^CD19^-^CD56^-^TCR_αβ_^+^TCR_γδ_^-^CD4^+^CD8^-^CD27^-^CD28^-^ |
| Naïve CD4 αβ T | CD14^-^CD19^-^CD56^-^TCR_αβ_^+^CD4^+^CD8^-^CD45RO^-^CCR7^+^ |
| Effector CD4 αβ T | CD14^-^CD19^-^CD56^-^TCR_αβ_^+^CD4^+^CD8^-^CD45RO^-^CCR7^-^ |
| Exhausted effector  CD4 αβ T | CD14^-^CD19^-^CD56^-^TCR_αβ_^+^CD4^+^CD8^-^CD45RO^-^CCR7^-^TIM-3^+^LAG-3^+^ |
| Central memory  CD4 αβ T | CD14^-^CD19^-^CD56^-^TCR_αβ_^+^CD4^+^CD8^-^CD45RO^+^CCR7^+^ |
| Exhausted central memory CD4 αβ T | CD14^-^CD19^-^CD56^-^TCR_αβ_^+^CD4^+^CD8^-^CD45RO^+^CCR7^+^TIM-3^+^LAG-3^+^ |
| Effector memory  CD4 αβ T | CD14^-^CD19^-^CD56^-^TCR_αβ_^+^CD4^+^CD8^-^CD45RO^+^CCR7^-^ |
| Exhausted effector memory CD4 αβ T | CD14^-^CD19^-^CD56^-^TCR_αβ_^+^CD4^+^CD8^-^CD45RO^+^CCR7^-^TIM-3^+^LAG-3^+^ |
| CD8 αβ T | CD3^+^CD14^-^CD19^-^CD56^-^TCR_αβ_^+^TCR_γδ_^-^CD4^-^CD8^+^ |
| Terminal effector  CD8 αβ T | CD3^+^CD14^-^CD19^-^CD56^-^TCR_αβ_^+^TCR_γδ_^-^CD4^-^CD8^+^CD25^-^CD69^+^ |
| Immediately activated  CD8 αβ T | CD3^+^CD14^-^CD19^-^CD56^-^TCR_αβ_^+^TCR_γδ_^-^CD4^-^CD8^+^CD27^-^CD28^-^ |
| Naïve CD8 αβ T | CD14^-^CD19^-^CD56^-^TCR_αβ_^+^CD4^-^CD8^+^CD45RO^-^CCR7^+^ |
| Effector CD8 αβ T | CD14^-^CD19^-^CD56^-^TCR_αβ_^+^CD4^-^CD8^+^CD45RO^-^CCR7^-^ |
| Exhausted effector  CD8 αβ T | CD14^-^CD19^-^CD56^-^TCR_αβ_^+^CD4^-^CD8^+^CD45RO^-^CCR7^-^TIM-3^+^LAG-3^+^ |
| Central memory  CD8 αβ T | CD14^-^CD19^-^CD56^-^TCR_αβ_^+^CD4^-^CD8^+^CD45RO^+^CCR7^+^ |
| Exhausted central memory CD8 αβ T | CD14^-^CD19^-^CD56^-^TCR_αβ_^+^CD4^-^CD8^+^CD45RO^+^CCR7^+^TIM-3^+^LAG-3^+^ |
| Effector memory  CD8 αβ T | CD14^-^CD19^-^CD56^-^TCR_αβ_^+^CD4^-^CD8^+^CD45RO^+^CCR7^-^ |
| Exhausted effector memory CD8 αβ T | CD14^-^CD19^-^CD56^-^TCR_αβ_^+^CD4^-^CD8^+^CD45RO^+^CCR7^-^TIM-3^+^LAG-3^+^ |
| CD8 γδ T | TCR_αβ_^-^TCR_γδ_^+^CD4^-^CD8^+^ |
| DN γδ T | TCR_αβ_^-^TCR_γδ_^+^CD4^-^CD8^-^ |
| ***PD-1^+^ cells*** |  |
| PD-1^+^ NK | CD3^-^CD14^-^CD19^-^CD56^+^PD-1^+^PD-L1^-^ |
| PD-1^+^ CD4 NKT | CD3^+^CD14^-^CD19^-^CD56^+^CD4^+^PD-1^+^PD-L1^-^ |
| PD-1^+^ CD8 NKT | CD3^+^CD14^-^CD19^-^CD56^+^CD4^-^PD-1^+^PD-L1^-^ |
| PD-1^+^ DC | CD3^-^CD14^-^CD19^-^CD56^-^CD11c^+^PD-1^+^PD-L1^-^ |
| PD-1^+^ monocyte | CD14^+^CD19^-^PD-1^+^PD-L1^-^ |
| PD-1^+^ B | CD14^-^CD19^+^PD-1^+^PD-L1^-^ |
| PD-1^+^ CD4 T | CD3^+^CD14^-^CD19^-^CD56^-^CD4^+^PD-1^+^PD-L1^-^ |
| PD-1^+^ CD8 T | CD3^+^CD14^-^CD19^-^CD56^-^CD4^-^PD-1^+^PD-L1^-^ |
| ***PD-L1+ cells*** |  |
| PD-L1^+^ NK | CD3^-^CD14^-^CD19^-^CD56^+^PD-1^-^PD-L1^+^ |
| PD-L1^+^ CD4 NKT | CD3^+^CD14^-^CD19^-^CD56^+^CD4^+^PD-1^-^PD-L1^+^ |
| PD-L1^+^ CD8 NKT | CD3^+^CD14^-^CD19^-^CD56^+^CD4^-^PD-1^-^PD-L1^+^ |
| PD-L1^+^ DC | CD3^-^CD14^-^CD19^-^CD56^-^CD11c^+^PD-1^-^PD-L1^+^ |
| PD-L1^+^ monocyte | CD14^+^CD19^-^PD-1^-^PD-L1^+^ |
| PD-L1^+^ B | CD14^-^CD19^+^PD-1^-^PD-L1^+^ |
| PD-L1^+^ CD4 T | CD3^+^CD14^-^CD19^-^CD56^-^CD4^+^PD-1^-^PD-L1^+^ |
| PD-L1^+^ CD8 T | CD3^+^CD14^-^CD19^-^CD56^-^CD4^-^PD-1^-^PD-L1^+^ |
| ***PD1^+^PD-L1^+^ cells*** |  |
| PD-1^+^PD-L1^+^ NK | CD3^-^CD14^-^CD19^-^CD56^+^PD-1^+^PD-L1^+^ |
| PD-1^+^PD-L1^+^ CD4 NKT | CD3^+^CD14^-^CD19^-^CD56^+^CD4^+^PD-1^+^PD-L1^+^ |
| PD-1^+^PD-L1^+^ CD8 NKT | CD3^+^CD14^-^CD19^-^CD56^+^CD4^-^PD-1^+^PD-L1^+^ |
| PD-1^+^PD-L1^+^ DC | CD3^-^CD14^-^CD19^-^CD56^-^CD11c^+^PD-1^+^PD-L1^+^ |
| PD-1^+^PD-L1^+^ monocyte | CD14^+^CD19^-^PD-1^+^PD-L1^+^ |
| PD-1^+^PD-L1^+^ B | CD14^-^CD19^+^PD-1^+^PD-L1^+^ |
| PD-1^+^PD-L1^+^ CD4 T | CD3^+^CD14^-^CD19^-^CD56^-^CD4^+^PD-1^+^PD-L1^+^ |
| PD-1^+^PD-L1^+^ CD8 T | CD3^+^CD14^-^CD19^-^CD56^-^CD4^-^PD-1^+^PD-L1^+^ |
| ***Regulatory cells*** |  |
| Foxp3^+^ CD4 T_reg_ | CD3^+^CD14^-^CD19^-^CD56^-^CD4^+^CD8^-^FoxP3^+^CD25^+^ |
| Foxp3^+^ CD8 T_reg_ | CD3^+^CD14^-^CD19^-^CD56^-^CD4^-^CD8^+^FoxP3^+^CD25^+^ |
| Foxp3^+^ CD8 NK_reg_ | CD3^-^CD14^-^CD19^-^CD56^+^CD4^-^CD8^+^FoxP3^+^CD25^+^ |
| Foxp3^+^ DN NK_reg_ | CD3^-^CD14^-^CD19^-^CD56^+^CD4^-^CD8^-^FoxP3^+^CD25^+^ |
| Foxp3^+^ CD4 NKT_reg_ | CD3^+^CD14^-^CD19^-^CD56^+^CD4^+^CD8^-^FoxP3^+^CD25^+^ |
| Foxp3^+^ CD8 NKT_reg_ | CD3^+^CD14^-^CD19^-^CD56^+^CD4^-^CD8^+^FoxP3^+^CD25^+^ |
| CTLA4^+^ CD4 T_reg_ | CD3^+^CD14^-^CD19^-^CD56^-^CD4^+^CD8^-^CTLA4^+^CD25^+^ |
| CTLA4^+^ CD8 T_reg_ | CD3^+^CD14^-^CD19^-^CD56^-^CD4^-^CD8^+^CTLA4^+^CD25^+^ |
| CTLA4^+^ CD8 NK_reg_ | CD3^-^CD14^-^CD19^-^CD56^+^CD4^-^CD8^+^CTLA4CD25^+^ |
| CTLA4^+^ DN NK_reg_ | CD3^-^CD14^-^CD19^-^CD56^+^CD4^-^CD8^-^CTLA4^+^CD25^+^ |
| CTLA4^+^ CD4 NKT_reg_ | CD3^+^CD14^-^CD19^-^CD56^+^CD4^+^CD8^-^CTLA4^+^CD25^+^ |
| CTLA4^+^ CD8 NKT_reg_ | CD3^+^CD14^-^CD19^-^CD56^+^CD4^-^CD8^+^CTLA4^+^CD25^+^ |
| CTLA4 in FoxP3^+^ CD4 Treg | CD3^+^CD14^-^CD19^-^CD56^-^CD4^+^CD8^-^FoxP3^+^CD25^+^CTLA4^+^ |
| HLA-DR^-^ MDSC | CD3^-^CD14^+^CD19^-^CD56^-^CD11b^+^HLA-DR^-^ |
| HLA-DR^dim^ MDSC | CD3^-^CD14^+^CD19^-^CD56^-^CD11b^+^HLA-DR^dim^ |
| MHC II^+^ NK | CD3^-^CD14^-^CD19^-^CD56^+^MHC II^+^ |
| MHC II^+^ CD4 NKT | CD3^+^CD14^-^CD19^-^CD56^+^CD4^+^MHC II^+^ |
| MHC II^+^ CD8 NKT | CD3^+^CD14^-^CD19^-^CD56^+^CD4^-^MHC II^+^ |
| MHC II^+^ CD4 T | CD3^+^CD14^-^CD19^-^CD56^-^CD4^+^MHC II^+^ |
| MHC II^+^ CD8 T | CD3^+^CD14^-^CD19^-^CD56^-^CD4^-^MHC II^+^ |
| ***PD-1^+^ Regulatory cells*** |  |
| PD-1^+^ Foxp3^+^ CD4 T_reg_ | CD3^+^CD14^-^CD19^-^CD56^-^CD4^+^CD8^-^FoxP3^+^CD25^+^PD-1^+^ |
| PD-1^+^ Foxp3^+^ CD8 T_reg_ | CD3^+^CD14^-^CD19^-^CD56^-^CD4^-^CD8^+^FoxP3^+^CD25^+^PD-1^+^ |
| PD-1^+^ Foxp3^+^ CD8 NK_reg_ | CD3^-^CD14^-^CD19^-^CD56^+^CD4^-^CD8^+^FoxP3^+^CD25^+^PD-1^+^ |
| PD-1^+^ Foxp3^+^ DN NK_reg_ | CD3^-^CD14^-^CD19^-^CD56^+^CD4^-^CD8^-^FoxP3^+^CD25^+^PD-1^+^ |
| PD-1^+^ Foxp3^+^ CD4 NKT_reg_ | CD3^+^CD14^-^CD19^-^CD56^+^CD4^+^CD8^-^FoxP3^+^CD25^+^PD-1^+^ |
| PD-1^+^ Foxp3^+^ CD8 NKT_reg_ | CD3^+^CD14^-^CD19^-^CD56^+^CD4^-^CD8^+^FoxP3^+^CD25^+^PD-1^+^ |
| PD-1^+^ CTLA4^+^ CD4 T_reg_ | CD3^+^CD14^-^CD19^-^CD56^-^CD4^+^CD8^-^CTLA4^+^CD25^+^PD-1^+^ |
| PD-1^+^ CTLA4^+^ CD8 T_reg_ | CD3^+^CD14^-^CD19^-^CD56^-^CD4^-^CD8^+^CTLA4^+^CD25^+^PD-1^+^ |
| PD-1^+^ CTLA4^+^ CD8 NK_reg_ | CD3^-^CD14^-^CD19^-^CD56^+^CD4^-^CD8^+^CTLA4CD25^+^PD-1^+^ |
| PD-1^+^ CTLA4^+^ DN NK_reg_ | CD3^-^CD14^-^CD19^-^CD56^+^CD4^-^CD8^-^CTLA4^+^CD25^+^PD-1^+^ |
| PD-1^+^ CTLA4^+^ CD4 NKT_reg_ | CD3^+^CD14^-^CD19^-^CD56^+^CD4^+^CD8^-^CTLA4^+^CD25^+^PD-1^+^ |
| PD-1^+^ CTLA4^+^ CD8 NKT_reg_ | CD3^+^CD14^-^CD19^-^CD56^+^CD4^-^CD8^+^CTLA4^+^CD25^+^PD-1^+^ |

Abbreviation: DC, dendritic cell; DN, double negative; MHC II, major histocompatibility complex class II; NK, natural killer cell; NKT, natural killer T cell; PD-1, programmed cell death 1; PD-L1, programmed cell death ligand 1; TCR, T cell receptor.

**Supplementary Figure 1. Schematic illustration of lineage cell pedigree.**

We gated myeloid cells (CD45^+^) with CD45. Leukocytes can be sequentially divided into B cells (CD14^-^CD19^+^), monocytes (CD14^+^CD19^-^), natural killer cells (NK, CD14^-^CD19^-^CD3^-^CD56^+^), NKT cells (CD14^-^CD19^-^CD3^+^CD56^+^), T cells (CD14^-^CD19^-^CD3^+^CD56^-^), and dendritic cells (DC, CD14^-^CD19^-^CD3^-^CD56^-^CD11c^+^). CD4 (CD4^+^) and CD8 (CD8^+^) NKT cells were identified by gating NKT cells with CD4 and CD8.

**Supplementary Figure 2. Schematic illustration for identifying granulocytes.**

CD11b^+^ cells (CD11b^+^lineage^-^) were gated myeloid cells with CD11b and lineage markers (CD3, CD14, CD19, CD56). CD11b^+^ cells can be sequentially divided into Basophil (CD123^+^CD66b^-^), eosinophil (CD123^-^CD66b^+^), and Neutrophil (CD66b^+^CD64^-^CD123^-^).

**Supplementary Figure 3. Schematic illustration for T-cell lineage.**

Leukocytes were divided into αβ T cells (TCRαβ^+^TCRγδ^-^), γδ T cells (TCRαβ^-^TCRγδ^+^), and double negative cells (DN, TCRαβ^-^TCRγδ^-^). αβ T cells can be divided into CD4 αβ T cells (CD4^+^CD8^-^), CD8 αβ T cells (CD4^-^CD8^+^), terminal effector CD4 αβ T cells (CD4^+^CD8^-^CD69^+^CD25^-^), and terminal effector CD8 αβ T cells (CD4^-^CD8^+^CD69^+^CD25^-^). γδ T cells were divided into DN γδ T (CD4^-^CD8^-^) cells and CD8 γδ T cells (CD4^-^CD8^+^). DN cells were used to identify CD8 NK cells (CD4^-^CD8^+^CD56^+^) and DN NK cells (CD4^-^CD8^-^CD56^+^).

**Supplementary Figure 4. Schematic illustration for T-cell lineage (continue).**

For identifying T-cell lineage, αβ T cells, gated from leukocytes, were divided into CD4 αβ T cells and CD8 αβ T cells. Then, naïve T cells (CCR7^+^CD45RO^-^), effector T cells (CCR7^-^CD45RO^-^), effector memory T cells (CCR7^-^CD45RO^+^), and central memory T cells (CCR7+CD45RO^+^) were sequentially identified from CD4 αβ T and CD8 αβ T subpopulation.

**Supplementary Figure 5. Schematic illustration for B-cell lineage.**

B cells (CD45^+^CD19^+^) were gated from leukocytes and subsequentially divided into IgG^+^ B cells, IgM^dim^ B cells, and IgM^+^ B cells. IgG^+^ B cells can be divided into long-lived plasma cells (CD10^+^CD21^+^), germinal center B cells (CD10^+^CD21^-^), and memory B cells (CD10^-^CD21^+^CD23^-^CD38^+^). IgM^dim^ B cells can be divided into short-lived plasma B cells (CD21^+^CD23^-^CD10^-^CD38^+^) and follicular B cells (CD21^+^CD23^-^CD10^-^CD38^+^). IgM+ B cells can be divided into marginal zone B cells (CD10^-^CD21^+^CD23^-^CD38^+^) and transition B cells (CD10^+^CD21^-^CD23^-^CD38^+^).
